# Treatment and Prevention of Post-dural Puncture Headaches: A Systematic Review

**DOI:** 10.7759/cureus.52330

**Published:** 2024-01-15

**Authors:** Rufaydah I Alatni, Rana Alsamani, Abdulelah Alqefari

**Affiliations:** 1 Anesthesia, Qassim University, Qassim, SAU; 2 Medicine and Surgery, Qassim University, Qassim, SAU

**Keywords:** lumbar puncture (lp), headache, epidural anaesthesia, spinal anesthesia, post dural puncture headache

## Abstract

Post-dural puncture headache (PDPH) is occasionally an inevitable side effect of neuraxial anesthesia, which can happen after spinal anesthesia or if an accidental dural puncture (ADP) happens during epidural anesthesia. The treatment and prevention options for PDPH differ widely from one institution to another. The management of PDPH is heterogeneous in many institutions because of the absence of clear guidelines and protocols for the management of PDPH. This study aimed to summarize all articles published during the past decade that discussed the treatment or prevention of PDPH. From 2013 to 2023, 345 publications were filtered for all treatment and prevention approaches used for PDPH patients. The Preferred Reporting Items for Systematic Review and Meta-Analyses (PRISMA) 2020 guidelines were followed for conducting this systematic review, and 38 articles were included for analysis and review. Existing data come from small randomized clinical trials and retrospective or prospective cohort studies. This review supports the effect of oral pregabalin and intravenous aminophylline in both treatment and prevention. Intravenous mannitol, intravenous hydrocortisone, triple prophylactic regimen, and neostigmine plus atropine combination showed effective and beneficial outcomes. On the other hand, neither neuraxial morphine nor epidural dexamethasone showed promising results. Consequently, the use of neuraxial morphine or epidural dexamethasone for the prevention of PDPH remains questionable. Regarding the posture of the patient and its consequences on the incidence of the headache, lateral decubitus is better than a sitting position, and a prone position is better than a supine position. Smaller non-cutting needles play a role in avoiding PDPH. Minimally invasive nerve blocks, including sphenopalatine ganglion or greater occipital nerves, are satisfyingly effective. Epidural blood patches remain the more invasive but the gold standard and ultimate solution in patients resisting medical therapy. This study highlights the need for larger research to define the best approach to prevent and treat PDPH.

## Introduction and background

Definition, risk factors, and clinical presentation

In the third edition of the International Classification of Headache Disorders (ICHD-3), the Headache Classification Committee of the International Headache Society (HIS) defines post-dural puncture headache (PDPH) as a headache developing within five days after a lumbar puncture and is attributed to cerebrospinal fluid (CSF) leakage through a dural hole, associated usually with a stiff neck and/or hearing symptoms [1]. Remission is spontaneous within two weeks if left untreated or after the sealing of the leak by an epidural blood patch (EBP) [1]. PDPH is a serious side effect of neuraxial anesthesia, which can happen after spinal anesthesia or if an accidental dural puncture (ADP) happens during epidural anesthesia. Female gender, youth, pregnancy, vaginal delivery, having a low body mass index, and not smoking are risk factors [2]. Patients with PDPH typically present with frontal or occipital headaches radiating to the neck or shoulder area within six to 72 hours of the procedure. The headache gets worse in an upright position and relieves in a supine position [2-4]. Nausea, vomiting, dizziness, tinnitus, stiff neck, and visual abnormalities are possible associated symptoms [5].

Incidence

The rate of unintentional puncture of the dura mater during epidural placement is 1.5% (95% CI: 1.5-1.5%), and over half of these patients (52.1%; 95% CI: 51.4-52.8%) experience PDPH [6]. Additionally, 76-85% of patients may develop PDPH according to a more recent study [7]. However, PDPH is more frequently caused by dural puncture during epidural anesthesia than by spinal anesthesia because spinal anesthesia uses small, pencil-point needles. When a pencil-point spinal needle is used, the risk of PDPH is reduced [2]. The risk of PDPH can be influenced by the size, shape, and orientation of the spinal needles, as well as the patient's posture [4].

Pathophysiology

CSF leakage from the dura, which leads to traction on pain-sensitive structures, is the cause of PDPH [3]. The CSF is a clear fluid produced in the choroid plexus inside the ventricles of the brain and reabsorbed by arachnoid granulations of the arachnoid matter into the bloodstream [3]. The average CSF volume in an adult is 150 mL, filling the cranial and spinal cavities [3]. If there is CSF leak as a consequence of a dural perforation significant enough to exceed CSF production, the CSF pressure will drop, and CSF hypotension occurs [3]. It is expected that, if more than 10% of the total CSF volume is lost, orthostatic headache will develop [3]. There are two proposed mechanisms explaining how headaches are brought on by CSF hypotension. The sagging theory claims that, when the patient takes an upright position, the reduced volume of CSF will be pulled down and shifted from the cranial cavity to the vertebral canal [3]. Hence, the brain sags into the foramen magnum with the meninges and cranial nerve being pulled consecutively [3]. This theory explains the symptoms of cranial nerve palsies seen in some patients with PDPH [3]. The second proposed theory states that cerebral vasodilation, which will occur to compensate for CSF loss and to maintain a constant total intra-cranial volume, is the reason for the headache [3].

The treatment and prevention options for PDPH differ widely from one institution to another [8]. The management of PDPH is heterogeneous in many institutions because of the absence of clear guidelines and protocols for the management of PDPH [8]. The currently available treatment options in the literature are bed rest, acetaminophen, caffeine, pregabalin, aminophylline, hydrocortisone, mannitol, neostigmine plus atropine, cosyntropin, sphenopalatine ganglion and greater occipital nerve blocks, and the more invasive EBP [3,8,9]. The greater occipital nerve supplies the skin over the posterior scalp up to the coronal suture. This nerve can be blocked medial to the occipital artery and lateral to the nuchal midline. Greater occipital nerve block will omit sensation from skin, muscles, and vasculature over the posterior side of the head [3]. The sphenopalatine ganglion is composed of sympathetic, parasympathetic, and sensory fibers [3]. It is located in the posterior nasal pharynx in the pterygopalatine fossa [3]. It can be blocked trans-nasally using cotton-tipped applicators soaked in lidocaine [3]. EBP has been suggested to be a useful treatment for severe or incapacitating PDPH, as well as a preventive measure for high-risk individuals. However, due to its invasiveness, requirement for anesthesiologists, and doubtful effectiveness, there are a number of issues with its application [5]. The incidence can be greatly decreased by paying attention to procedure-related factors. The position of the patient during the procedure and the size and shape of the needle all seem to play a role in the prevention of PDPH.

Subdural hematoma, diplopia as a result of cranial nerve palsy, cerebral venous thrombosis, chronic headache, and post-partum depression have been reported as complications of unintentional dural puncture (UDP) [3]. PDPH is occasionally an inevitable side effect. As a result, anesthesiologists must understand prevention and treatment strategies. This study aimed to summarize all articles published during the past decade that discussed the treatment or prevention of PDPH.

## Review

Methods

We followed the Preferred Reporting Items for Systematic Review and Meta-Analyses (PRISMA) 2020 guidelines for conducting this systematic review [10]. PubMed and ScienceDirect were explored for studies published between 2013 and 2023. The search strategy included the following keywords: ((post-dural puncture headache [Title/Abstract]) AND (treatment [Title/Abstract])). The search strategy was not limited by geographical criteria. Only English-language articles were reviewed for inclusion. Both peer-reviewed experimental and observational studies were included. After the identification process, two independent co-authors screened the information from the publications based on the title and abstract. The initial analysis of the two databases resulted in 345 publications. After further elimination according to the below criteria, a total of 38 were found that covered the aim of this review.

Inclusion Criteria

This review included full-text publications that focused on treatment and/or preventive measures of PDPH, published between 2013 and 2023 and written in English.

Exclusion Criteria

**Figure 1:**
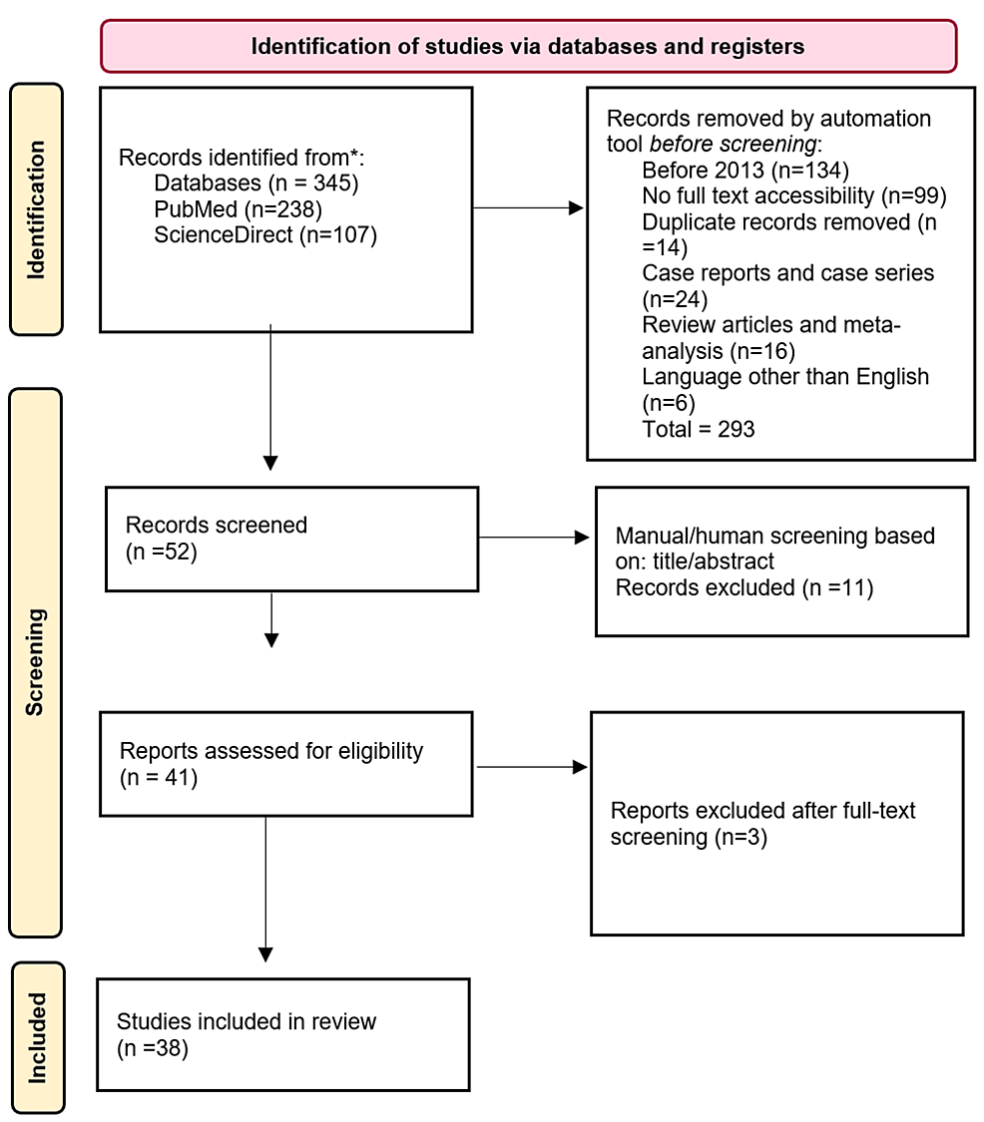
PRISMA 2020 flow diagram illustrating the inclusion and exclusion process.

Review articles, case reports, and case series were not considered. Duplicate articles were excluded. The inclusion and exclusion methods of this review are shown in Figure 1.

Bias

Using the Grading of Recommendations, Assessment, Development, and Evaluation (GRADE) scale, each article included in this review was evaluated separately for the risk of bias. The overall bias in this review is minimal.

Results

During the identification phase, a total of 345 papers were attained. Among them, 238 were from PubMed, and 107 were from ScienceDirect. Automatic screening using the Rayyan bibliographic software resulted in the exclusion of 293 articles, leading to 52 articles for manual screening. Specifically, 14 were duplicates, 134 were published before 2013, 99 were not found as full texts, 24 were case reports, 16 were review articles, and six were excluded because they were not written in English. Fifty-two publications were manually assessed through the previously mentioned inclusion and exclusion criteria, and 41 publications were full-text analyzed. Ultimately, 38 publications were included. All publications used in this review are summarized in Table 1.

**Table 1:** Summary of publications used to generate the data of the systematic review via PRISMA guidelines [10]. PDPH: post-dural puncture headache; VAS: visual analog scale; IV: intravenous; ADP: accidental dural puncture; EBP: epidural blood patch; TLP: traumatic lumbar puncture; ALL: acute lymphoblastic leukemia; HES: hydroxyethyl starch; SIH: spontaneous intracranial hypotension; INR: international normalized ratio; CSF: cerebrospinal fluid; NRS: numeric rating scale; GONB: greater occipital nerve block; SPGB: sphenopalatine ganglion block

S. no	Author	Country	Design and study population	Findings	Conclusion
1	Neuman et al. (2013) [11]	USA	Retrospective assessment of medical records (n=285)	79% of PDPH patients were successfully managed with conservative therapies alone (bedrest, IV fluids, analgesics, antiemetics), on the other hand, 21% required progression to interventional therapies (epidural blood or fibrin glue patch procedures) for full resolution of symptoms.	The majority of the participants had self-limited symptoms that were alleviated with conservative medical therapy. For the remaining PDPH patients who were not responding to conservative therapy, epidural blood patches or the application of epidural fibrin glue proved to be therapeutically successful options.
2	Bakshi et al. (2018) [12]	India	A retrospective analysis (n=407)	All patients were given pharmacological treatment. 71% of patients were either on coffee or caffeine tablets. One case of persistent PDPH showed a good response to oral pregabalin 75 mg.	PDPH can be effectively controlled with drug treatment only.
3	Mahoori et al. (2014) [13]	Iran	Randomized trial (n=90)	The pregabalin group had a considerably lower mean VAS score than the other groups, and the gabapentin group had a lower mean VAS score than the acetaminophen group.	Both pregabalin and gabapentin showed a noticeable effect in the treatment of PDPH. But, pregabalin is more effective.
4	Karami et al. (2021) [14]	Iran	Randomized controlled trial (n=136)	The rate of PDPH was significantly less in the intervention group compared to the placebo group.	Oral pregabalin had a preventive effect on PDPH when given one night before spinal anesthesia.
5	Nofal et al. (2014) [15]	Egypt	Double-blind, randomized, placebo-controlled study. (n=88)	In both gabapentin and placebo groups, the frequency of headache and associated symptoms was similar. In the gabapentin group, headache onset was noticeably delayed. The intensity and duration of headaches were also significantly reduced in the gabapentin group.	No effect was found of pre-operative gabapentin on the incidence of PDPH. Gabapentin delays the onset and reduces the severity and duration of PDPH in parturients undergoing spinal anesthesia.
6	Razavizadeh et al. (2022) [16]	Iran	Randomized clinical trial (n=180)	The placebo group had the highest headache Severity. and the lowest headache severity in the aminophylline group. The dexamethasone group also experienced less pain severity than the placebo group but more than the aminophylline group, and these differences were significant.	Headache after spinal anesthesia was significantly reduced after intravenous administration of aminophylline and dexamethasone. In addition, those receiving aminophylline experienced noticeably less severe headaches than those receiving dexamethasone.
7	Yang et al. (2019) [17]	China	Randomized controlled trial (n=120)	The incidence of PDPH in the aminophylline group was significantly lower than in the normal saline group.	Intraoperative intravenous infusion of 250 mg aminophylline was associated with a lower incidence of PDPH after caesarean section with no side effects.
8	Wu et al. (2016) [18]	China	Prospective evaluation (n=32)	Thirty minutes after starting IV aminophylline, over 50% of the patients said they were "very much improved" or "much improved," and two days later, 93% of the patients said the same.	Intravenous injection of aminophylline is probably an effective and safe early-stage treatment for PDPH.
9	Naghibi et al. (2014) [19]	Iran	Randomized controlled trial (n=140)	Patients who received a combination of aminophylline plus dexamethasone had significantly less incidence of PDPH than patients who received aminophylline alone or dexamethasone alone.	The combination of administering 1.5 mg/kg of aminophylline and 0.1 mg/kg of dexamethasone significantly decreased PDPH better than using either drug separately in patients undergoing lower extremities surgery under spinal anesthesia.
10	Najafi et al. (2014) [20]	Iran	Clinical trial (n=268)	The overall incidence of headache as well as the severity of it showed no statistically significant difference in cases versus in controls.	This clinical trial did not find any significant preventive effect of epidural injection of dexamethasone in cases with PDPH, In contrast to previous research that demonstrated the benefit of intravenous dexamethasone in the prevention and treatment of PDPH.
11	Shahriari et al. (2021) [21]	Iran	Single-blind randomized clinical trial (n=80)	Both of mannitol group and the acetaminophen-caffeine group had a significant reduction in the pain scores after treatment. However, mannitol administration was more effective than acetaminophen-caffeine in pain reduction and yielded higher patient satisfaction than the caffeine group in patients undergoing spinal anesthesia.	Compared to acetaminophen-caffeine capsules, IV mannitol could be more effective in treating PDPH.
12	Kassim et al. (2016) [9]	Egypt	A randomized double-blind study (n=50)	The VAS score was significantly lower in the hydrocortisone group compared to the mannitol group at 6, 12, and 24 h. However, at 48 h, both groups had nearly equal scores of headache intensity.	Both intravenous hydrocortisone and mannitol intravenous infusion were successful in reducing PDPH within 48h. In fact, hydrocortisone was earlier in the relief of headaches.
13	Riveros Perez et al. (2020) [22]	USA	Retrospective evaluation (n=31)	Among the first group of 14 cases received triple prophylaxis, three patients developed PDPH (21%), with two of them requiring a blood patch (14%). The second group of nine patients who underwent measures different than triple prophylaxis had a PDPH rate of 55% and only one patient required a blood patch (11%).	In obstetric patients with ADP, the triple prophylactic regimen which consisted of epidural saline, IV cosyntropin, and epidural morphine succeeded in reducing the incidence of PDPH and the need for blood patches.
14	Brinser et al. (2019) [23]	USA	Retrospective cohort study (n=80)	38 women received neuraxial morphine (group 1) and 42 did not (group 2). Upon comparing the outcome between the two groups, there was no significant difference regarding the incidence of headaches nor the need for EBP or the headache intensity. Furthermore, the morphine group had a higher hospital length of stay.	Neuraxial morphine may not have a preventive role against the risk of PDPH in cases of ADP.
15	Peralta et al. (2020) [24]	USA	A randomized double-blind trial (n=61)	No differences were documented between groups regarding the onset, duration, severity of headache, or presence of cranial nerve symptoms. The incidence of PDPH in the intrathecal morphine group was 78%, and 79% in the intrathecal saline group.	This research challenges the efficacy of prophylactic intrathecal morphine after accidental dural puncture.
16	Ahmadzade et al. (2023) [25]	Iran	A randomized, controlled, double‑blind clinical trial (n=99)	The frequency of headaches in the control group was higher than in the study group. 62% of participants in the control group and 40% in the study group developed headaches with a PDPH profile during 5 days of follow‑up. In the study group, 2 patients needed drug treatment to control headaches. On the other hand, 26 out of 31 patients in the control group needed drugs to control headaches.	Preventive administration of a combination of 40 μg/kg of neostigmine plus 20 μg/kg of atropine may be useful in reducing the severity and frequency of PDPH after spinal anesthesia in lower limb orthopedic surgeries.
17	Sharma et al. (2022) [26]	India	A randomized controlled trial (n=134)	In the sitting position group, 17.91% developed PDPH. In the lateral recumbent posture group, only 4.48% developed PDPH.	This study concludes that the lateral recumbent posture is better than the sitting posture for administering spinal anesthesia as it is associated with lower incidence and severity of PDPH.
18	Alizadeh et al. (2022) [27]	Iran	Cohort (n= 1416)	Among patients with pilonidal who were operated in the prone position, 0.68% experienced headaches. On the other hand, 8.95% of those operated in supine positions got headaches.	Prone position during surgery is associated with lower frequency of PDPH in patients with spinal or epidural anesthesia.
19	Engedal et al. (2015) [28]	Denmark	Prospective interventional trial (n=501)	After the use of smaller non-cutting needles, there were notable decreases in the incidence of PDPH, days off from work, hospital stays, and blood patch treatments. Additionally, during the process, there was a decrease in the number of failed attempts and the first operator's failure rate.	Smaller, non-cutting needles decrease the incidence of PDPH. In addition, they are easier to use in outpatient clinics and by changing the needle, procedural difficulty and overall costs were decreased.
20	Långström et al. (2022) [29]	Finland	Prospective single-arm study (n=50)	The success of the first attempt was 79.5%, with the CSF detection sensitivity of 86.1%. TLP incidence was 17.3%. Six percent of patients experienced post-dural puncture headaches in the week after the treatment. There were no significant complications noted throughout the follow-up.	In a real-world clinical context, the innovative bioimpedance spinal needle system demonstrated a high success rate and a low incidence of TLP and other complications among pediatric ALL patients, suggesting the system's possible application in pediatric hemato-oncology.
21	Pirbudak et al. (2014) [30]	Turkey	Retrospective study (n=77)	For female patients, the mean headache duration was 3.1 ± 1.3 days, and for male patients, it was 4.6 ± 2.3 days (p=0.020). 10 minutes after EBP administration, patients saw a significant drop in VAS and a significant rise in patient satisfaction (p=0.001). When EBP was administered, 17 participants (22.07%) developed transient radicular pain.	EBP is an effective and generally safe technique in PDPH cases, particularly for obstetric patients. We noted that in female patients, symptoms of PDPH appeared earlier. Small diameter needles (less than 22 G) and the avoidance of repeated attempts were shown to be important for spinal anesthesia.
22	Gupta et al. (2020) [31]	International study	A prospective, cohort study (n=1001)	1001 patients in total were included, representing 24 nations; of these, 64.6% had an EBP and 35.4% did not. A higher initial headache intensity was linked to a higher EBP utilization rate. Four hours after EBP, the intensity of headaches decreased significantly, and 19.3% of patients had another EBP. Seven days following the diagnosis, there were often no or very minor headaches. At three months, the EBP group experienced more episodes of headache, back pain, and painkiller use.	Although management practices vary between countries, patients with more initial intense headaches were more likely to receive EBP. EBP rapidly decreased the intensity of headaches; however, 20% of patients required a second EBP. Most patients experienced little to no headaches after seven days.
23	Stein et al. (2014) [32]	USA	A prospective randomized controlled study (n=109)	18.3% of patients in the prophylactic epidural blood patch group developed PDPH compared with 79.6% in the therapeutic EBP group (p<0.0001).	An epidural blood patch is an effective way to prevent and reduce the incidence of PDPH in obstetric patients.
24	Tang et al. (2023) [33]	China	Retrospective study (n=85)	The rates of PDPH were 84%, 52.6%, and 54.5% with conservative, prophylactic EBP, and prophylactic epidural hydroxyethyl starch (HES), respectively. Prophylactic EBP and prophylactic epidural HES therapy greatly reduced the incidence of PDPH when compared to the conservative treatment. Therapeutic EBP was utilized much less in the prophylactic EBP and prophylactic epidural HES groups than in the conservative therapy group. Prophylactic EBP considerably shortened the length of hospital stay while prophylactic epidural HES showed no statistical difference compared with conservative treatment.	The incidence of PDPH can be minimized by using preventive therapy with EBP and epidural HES infusion. Prophylactic EBP considerably shortened the length of hospital stay.
25	Lee et al. (2021) [34]	South Korea	Retrospective study (n=68)	The number of patients needing repeated EBPs was significantly higher in the spontaneous intracranial hypotension (SIH) group compared to the PDPH group (P = 0.007). Forty patients (90.9%) and 17 patients (70.8%) achieved complete recovery from headache after a single epidural blood patch in the PDPH group and SIH group, respectively (P < 0.001).	Most patients in the PDPH group required a single EBP to achieve complete recovery from headaches. Contrarily, patients in the SIH group required repeated epidural blood patches for complete pain relief.
26	Lee et al. (2021) [35]	South Korea	Retrospective study (n=105)	Patients with SIH required more epidural blood patch treatment than those with PDPH. The SIH group included a higher proportion of patients who underwent repeated EBP treatment.	Patients with SIH required more than one EBP treatment when compared to patients with PDPH.
27	Oh et al. (2022) [36]	South Korea	Retrospective cohort (n=596)	Patients who needed repeated EBPs were 21.1%. 34.5% in SIH, and 9.2% in the iatrogenic population. CSF leakage on myelographies and the INR in patients with SIH consistently showed significant associations with repeated EBPs.	Patients with SIH may require repeated EBPs more frequently. In patients with SIH, prolonged INR and CSF leakage have been associated with repeated EBPs. To identify the variables linked to recurring EBP requirements, more research is required.
28	Gupta et al. (2022) [37]	International study	Prospective, multicenter, international cohort study (n=591)	Data to classify failure were available in 591 patients. 167 patients (28.3%) had a failed epidural blood patch; 195 patients (34.0%) had a successful outcome, and 229 patients (38.7%) had partial success. Patients with a history of migraine showed a statistically significant correlation with failure when the ADP occurred between lumbar levels L1/L3 compared with L3/L5 and when an epidural blood patch was performed in a short time after the ADP.	28.3% of women had a failed epidural blood patch. factors associated with failed EBP were a shorter time between an accidental dural puncture and an epidural blood patch, as well as a higher lumbar level of the accidental puncture. a history of migraine increases the risk of a second EPB.
29	Dupoiron et al. (2021) [38]	USA	Retrospective cohort study (n=199)	The incidence of PDPH is significantly reduced by fibrin glue application, going from 32.7% in the no-glue group to 10.92% (P < 0.001) in the glue group. Following the administration of fibrin glue, no severe PDPHs were observed. Additionally, the fibrin glue group's maximal symptom duration was statistically shorter (3 days) than the no-glue group's (15 days).	The recent use of fibrin glue seems promising regarding its impact on PDPH and its safety profile. Also, its affordable cost and reproducibility make it an efficient option.
30	Youssef et al. (2021) [39]	Egypt	A randomized clinical trial (n=93)	There was a significant difference between groups after 2 hours in supine and sitting headache Numeric Rating Scale (NRS). However, both treatments showed similar effectiveness from the third hour afterwards.	Both GONB and SPGB are equally successful and effective in treating PDPH symptoms. compared to EBP, both procedures are more safe, easier, and less invasive.
31	Gayathri et al. (2022) [40]	India	A randomized control study (n=40)	The group of patients who had SPG block had significantly lower headache pain scores and less total paracetamol consumption. furthermore, a markedly better satisfaction score was reported in the study group. One patient in the control group needed an epidural blood patch.	SPG block is a good alternative in treating PDPH. the requirement for an epidural blood patch is markedly reduced with the implication of SPG block. Fast and sustained pain relief as well as procedural safety make it an evolving management for PDPH.
32	Puthenveettil et al. (2018) [41]	India	A prospective unblinded observational study (n=20)	Within five minutes following the block, 88.89% of patients in group B who received SPGB experienced sufficient pain alleviation. For up to eight hours, Group B experienced much less pain with no side effects.	SPGB is among the effective initial modalities for treating severe PDPH.
33	Nazir et al. (2021) [42]	IND	Single-blinded randomized study (n=20)	In the first 24 hours following the SPG block, patients in group 1(applicator group) saw a statistically significant decrease in their VAS score in comparison to group 2 (nasal spray group). After that, until discharge, the groups' pain scores were similar.	For PDPH, the trans-nasal SPG block represents a minimally invasive therapy approach that eliminates the need for more invasive procedures. The applicator technique of a trans-nasal SPG block produces superior pain alleviation compared to the nasal spray approach.
34	Jespersen et al. (2020) [43]	Denmark	A randomized, blinded, clinical trial (n=40)	40 patients were randomized with a baseline upright median pain intensity of 74 and 84 mm in the local anaesthetic and placebo groups, respectively. At 30 min after sphenopalatine ganglion block, the median pain intensity in an upright position was 26mm in the local anaesthetic group versus 37mm in the placebo group. 45% of the placebo group and 50% of the local anesthetic group needed an EBP.	No statistically significant difference was evident between the administration of a sphenopalatine ganglion block with local anesthetic and placebo.
35	Azzi et al. (2022) [44]	Lebanon	Retrospective case–control (n=90)	Seven patients out of 18 (38.89%) had their headaches relieved on conservative treatment only. Six (33.33%) of the 11 patients who remained had their symptoms resolved on GONB, and their pain score had significantly decreased 48 hours after GONB compared to baseline. A blood patch was used to treat the symptoms of five patients (27.78%), and the pain score significantly decreased following the blood patch as compared to the baseline.	According to our preliminary findings, ultrasound-guided GONB is a relatively safe and effective option for treating patients who are unresponsive to conservative therapy.
36	Uyar Türkyilmaz et al. (2016) [45]	Turkey	Retrospective study (n=16)	The mean VAS score of the patients before the block was 8.75 (±0.93); after that, it decreased to 3.87 (±1.78) 10 min after the block and then to 1.18 (±2.04) 2 h after the block and to 2.13 (±1.64) 24 h after the block.	GON block was shown to be an effective, minimally invasive, and easy method in the treatment of PDPH especially after caesarean operations. It could be taken into consideration before the application of a blood patch.
37	Akyol et al. (2015) [46]	Turkey	Retrospective study (n=21)	Mean VAS pain scores at 10 minutes and 6, 10, 15, and 24 hours following the block were considerably improved.	An ultrasound-guided bilateral occipital nerve block may be effective for PDPH patients who do not respond to conventional medicinal treatment.
38	Abdelraouf et al. (2019) [47]	Egypt	A randomized-controlled trial (n=90)	The study group reported a lower headache score compared to the control group at all the post-injection time points. Time to the first analgesic request was delayed in group S compared to group C. All patients in group C required rescue analgesia, whereas only 6 (13.3%) patients in group S asked for an analgesic.	Ultrasound-guided injection of the dexamethasone-lidocaine combination in suboccipital muscles is an effective modality of treatment in patients with PDPH after cesarian section.

Discussion

Pharmacological Treatment

This systematic review identified 16 publications assessing medical drugs for the treatment and prevention of PDPH [9,11-25]. According to a retrospective assessment of medical records that included 285 patients and was published in 2013, analgesics and antiemetics, along with bed rest and IV fluids, were sufficient to manage the symptoms of PDPH in about 79% of patients [11]. Another retrospective assessment of 407 cases concluded that PDPH can be effectively controlled with drug treatment only, mostly caffeine tablets [12]. Among antiepileptic medications, gabapentin and pregabalin are well-known to play a role in pain alleviation for patients with PDPH. Three randomized control studies investigated the role of antiepileptic drugs in the treatment or prevention of PDPH [13-15]. In one Irani study, the researchers compared the effect of pregabalin, gabapentin, and acetaminophen in patients with PDPH. Interestingly, the visual analog scale (VAS) score was the highest in the acetaminophen group than in the gabapentin group and the lowest in the pregabalin group, concluding that pregabalin is the most effective medication in that trial [13]. The remaining two trials discussed the prevention of PDPH [14,15]. Oral pregabalin administered one night before spinal anesthesia was associated with a decreased incidence of PDPH compared to placebo [14]. On the other hand, pre-operative gabapentin had no significant effect on the incident in an Egyptian study; however, its beneficial effect in reducing the severity and the duration, as well as delaying the onset of the headache, was reported [15].

Intravenous aminophylline is successful in both treatment and prevention of PDPH [16-19]. In one study, the combination of 0.1 mg/kg of dexamethasone and 1.5 mg/kg of aminophylline enhanced the effect of aminophylline when compared to dexamethasone alone and aminophylline alone [19]. The role of dexamethasone alone in the treatment or prevention of PDPH seems to be questionable. A clinical trial published in 2014 denied the prophylactic effect of epidural dexamethasone in PDPH cases as there was no statistical difference between cases and controls [20]. However, in a more recent paper published in 2022, there was an association between intravenous dexamethasone administration and better outcomes in pain alleviation compared to placebo [16].

Mannitol intravenously was shown to be more effective in treating PDPH than acetaminophen-caffeine capsules when these two methods were compared in a randomized clinical trial published in 2021 [21]. In another trial, intravenous mannitol was compared to intravenous hydrocortisone, which concluded that both medications are equally effective in pain relief; however, hydrocortisone had earlier onset [9].

The benefit of a triple prophylactic regimen consisting of epidural saline, morphine, and intravenous (IV) cosyntropin was evaluated in an observational study published in 2020 [22]. The group of patients who received a triple prophylactic regimen was compared to a group that received conservative measures, including oral paracetamol, oral ibuprofen, oral opioid-containing formulations, and intravenous caffeine. The triple prophylactic regimen succeeds in decreasing the rate of PDPH and the demand for blood patches. This was not the situation when morphine was administered alone intrathecally. In fact, two papers investigated the effectiveness of intrathecal morphine in reducing the incidence of headache after accidental dural puncture; in both papers, no statistically significant difference was documented between the study group and control group [23,24]. As a result, the clinical usefulness of neuraxial morphine in the prevention of PDPH is not supported by these findings [23,24].

A very recent clinical trial published in 2023 concluded that the combination of 40 μg/kg neostigmine plus 20 μg/kg of atropine is greatly effective in reducing the frequency of PDPH and the need for medical treatment to control the headache after spinal anesthesia [25]. Neostigmine is widely used in anesthesia practice as a reversal of the effect of non-depolarizing muscle relaxants. It is a quaternary amine cholinesterase inhibitor that increases CSF secretion. Despite the fact that anti‑choline esterase drugs generally increase levels of acetylcholine in neuromuscular junctions, and acetylcholine decreases CSF secretion in choroid plexuses, neostigmine competes with acetylcholine in entering the choroid plexus, which will the lower acetylcholine level and increase CSF secretion eventually [25]. Additionally, neostigmine is thought to be a cerebral vasoconstrictor by directly stimulating cerebrospinal ganglia, and this counteracts cerebral vasodilatation in PDPH and could be a possible explanation of the preventive effect of neostigmine in patients with PDPH. The beneficial effect of atropine in cases with PDPH can be explained by two actions, increasing CSF secretion by blocking the acetylcholine effect and cerebral vasoconstriction by the inhibition of sphenopalatine ganglion [25].

Position of the Patient

Spinal anesthesia can be administered in the sitting, lateral decubitus, or even prone position. Needless to say, each position has its own advantages and disadvantages. Besides all the previously mentioned factors that could influence the occurrence of PDPH, the position of the patient during the procedure influences the occurrence of PDPH as well [26]. Two recent papers published in 2022 discussed the impact of the posture on the incidence of PDPH [26,27]. The lateral decubitus position was shown to be better than the sitting position as it was associated with a lower incidence of PDPH [26]. A cohort study with a total of 1,416 patients compared between prone and supine position regarding the frequency of PDPH after the operation [27]. Moreover, 0.68% of the patients in the prone position group complained of having PDPH, whereas 8.95% of patients in the supine group experienced PDPH, concluding that the prone position is associated with a lower incidence of PDPH when compared to the supine position.

Needle Factors

We found three published papers evaluating the impact of the needle size on the incidence of headache after dural puncture and other associated complications [28-30]. A study conducted in Denmark concluded that smaller non-cutting needles significantly decreased the incidence of PDPH, as well as the first operator’s failure rate and the number of failed attempts [28]. The usage of smaller non-cutting needles in spinal anesthesia was also associated with a decrease in hospital stays, the number of days off from work, and the need for blood patch treatment [28]. A turkey study highlighted the benefit of a small diameter needle (less than 22 G) and the importance of avoiding multiple attempts in spinal anesthesia as it was linked to a lower incidence of headache after dural puncture according to their results [30]. The third article assessed the clinical application and performance of a novel bioimpedance spinal needle system. The study included 152 intrathecal treatment lumbar punctures (LP) done for 50 pediatric patients with acute lymphoblastic leukemia (ALL) [29]. The bioimpedance spinal needle system measures continuously the bioimpedance of tissues that are in immediate contact with the needle tip and gives an audio-visual alarm when the needle tip reaches CSF in the subarachnoid space [29]. The incidence of PDPH was 6% during the first week after the procedure, and no major complications were documented in the representative sample, concluding that the novel bioimpedance spinal needle system has achieved a high success rate. The promising results indicate clinical utility for the system in pediatric haemato-oncology [29].

Epidural Blood Patch

A total of 1,001 patients with PDPH from 24 countries were enrolled in an international cohort study to describe the management practices in PDPH cases and the effectiveness of EBP [31]. Variation was obvious between different countries regarding the management practices in cases of PDPH. However, EBP was the most chosen management in cases with higher initial headache severity [31]. Many other publications showed the efficacy and wide application of EBP in the treatment and prevention of PDPH [32-37]. A prospective study that began in 1997 and ended in 2005 declared that 18.3% of patients who received prophylactic EBP developed headaches eventually compared to 79.6% of the patient who did not receive EBP for prophylaxis, which highlights the preventive effect of EBP in PDPH cases [32].

Epidural infusion of hydroxyethyl starch (HES) was shown to have similar efficacy to prophylactic EBP in cases with unintentional dural puncture (UDP) [33]. However, prophylactic EBP was superior to prophylactic epidural HES in reducing the length of hospital stays of patients with UDP [33]. Three Korean retrospective studies compared the response to EBP between cases of PDPH and cases of spontaneous intracranial hypotension (SIH) [34-36]. In all three papers, patients with SIH required more epidural blood patch treatments and more often needed repeated epidural blood patch treatments compared to patients with PDPH [34-36]. The international normalized ratio (INR) was associated significantly with repeated EBPs in patients with SIH [36]. INR values were found to be high in SIH patients with poor response to EBP. Contrarily, INR was not a significantly associated factor with repeated EBP requirements in cases with iatrogenic injury [36]. However, patients with elevated INRs were not included in the study as they are not candidates for EBP due to the risk of complications such as hematoma formation. Thus, that paper cannot determine if improving the coagulation profile of patients will decrease the incidence of repeated EBPs [36]. CSF leakage was another factor associated with repeated EBP requirements in the representative sample [36]. Possible factors associated with failed EBPs are a shorter time between an accidental dural puncture and an epidural blood patch, as well as a higher lumbar level of the accidental puncture [37]. A history of migraine increases the risk of a second EPB [37].

Fibrin Glue Application

The novel use of Tisseel (fibrin glue) showed promising results [38]. A retrospective study included 199 patients matched to one of the two groups, with fibrin glue and without fibrin glue, and found that patients who received prophylactic fibrin glue had a shorter duration of symptoms than patients in the no-glue group [38]. Its impact on PDPH, its safety profile, its affordable cost, and its reproducibility make it an efficient technique [38].

Nerve Block

We reached eight publications that discussed nerve block as a treatment or preventive measure in PDPH [39-46]. An Egyptian randomized clinical trial demonstrated that sphenopalatine ganglion (SPG) block and greater occipital nerve block (GONB) are equally effective in the treatment of PDPH [39]. Moreover, both approaches are less invasive and safer than EBP [39]. Two Indian publications support SPG block as a good alternative modality in treating PDPH [40,41]. A single-blinded randomized study highlighted that the applicator technique of a trans-nasal SPG block produces superior pain alleviation compared to the nasal spray approach [42]. Contrarily, one trial revealed no significant difference between SPG block and placebo in terms of PDPH treatment [43]. Three retrospective studies evaluated the efficacy of GONB in the treatment of PDPH [44-46]. Ultrasound-guided GONB has shown to be a good option for patients not responding to conservative therapy and could be taken into consideration before the application of a blood patch [44-46]. An Egyptian randomized controlled trial investigated the efficacy of injecting dexamethasone plus lidocaine in the suboccipital muscles to relieve the headache after spinal anesthesia in women undergoing cesarian section [47]. The group of women who had ultrasound-guided injection of dexamethasone plus lidocaine in the suboccipital muscle reported lower headache scores compared to the control group at all the post-injection time points, concluding that this approach is effective in treating PDPH [47].

Limitations

Only a few publications covered each modality of PDPH management, although that paints a picture of the current protocol in managing PDPH, which was a goal.

## Conclusions

PDPH is one of the most common complications of spinal anesthesia and accidental dural puncture during epidural anesthesia. This systematic review summarizes all articles published in the past decade that discussed the treatment or prevention of PDPH. Existing data come from small randomized clinical trials and retrospective or prospective cohort studies. This review supports the effect of oral pregabalin and intravenous aminophylline in both treatment and prevention. Intravenous mannitol, intravenous hydrocortisone, triple prophylactic regimen, and neostigmine plus atropine combination showed effective and beneficial outcomes. On the other hand, neither neuraxial morphine nor epidural dexamethasone showed promising results. Consequently, the use of neuraxial morphine or epidural dexamethasone for the prevention of PDPH remains questionable. Regarding the posture of the patient and its consequences on the incidence of the headache, lateral decubitus is better than a sitting position, and a prone position is better than a supine position. Smaller non-cutting needles play a role in avoiding PDPH. Minimally invasive nerve blocks including sphenopalatine ganglion or greater occipital are satisfyingly effective. Epidural blood patches remain the more invasive but the gold standard and ultimate solution in patients resisting medical therapy. Larger research is warranted to define the best approach to prevent and treat PDPH.
